# Hyperuricemia and Risk of Incident Hypertension: A Systematic Review and Meta-Analysis of Observational Studies

**DOI:** 10.1371/journal.pone.0114259

**Published:** 2014-12-01

**Authors:** Ji Wang, Tianqiang Qin, Jianrong Chen, Yulin Li, Ling Wang, He Huang, Jing Li

**Affiliations:** 1 Department of Evidence-Based Medicine and Clinical Epidemiology, West China Hospital, Sichuan University, Chengdu, China; 2 Second Affiliated Hospital of Chengdu University of TCM, Chengdu University of TCM, Chengdu, China; 3 Department of Cardiology, Mianyang People's Hospital, Mianyang City, China; 4 Department of Cardiology, West China Hospital, Sichuan University, Chengdu, China; Mario Negri Institute for Pharmacological Research and Azienda Ospedaliera Ospedali Riuniti di Bergamo, Italy

## Abstract

**Background:**

Observational studies of the relationship between hyperuricemia and the incidence of hypertension are controversial. We conducted a systematic review and meta-analysis to assess the association and consistency between uric acid levels and the risk of hypertension development.

**Methods:**

We searched MEDLINE, EMBASE, CBM (Chinese Biomedicine Database) through September 2013 and reference lists of retrieved studies to identify cohort studies and nested case-control studies with uric acid levels as exposure and incident hypertension as outcome variables. Two reviewers independently extracted data and assessed study quality using Newcastle-Ottawa Scale. Extracted information included study design, population, definition of hyperuricemia and hypertension, number of incident hypertension, effect sizes, and adjusted confounders. Pooled relative risks (RRs) and corresponding 95% confidence intervals (CIs) for the association between hyperuricemia and risk of hypertension were calculated using a random-effects model.

**Results:**

We included 25 studies with 97,824 participants assessing the association between uric acid and incident hypertension in our meta-analysis. The quality of included studies is moderate to high. Random-effects meta-analysis showed that hyperuricemia was associated with a higher risk of incident hypertension, regardless of whether the effect size was adjusted or not, whether the data were categorical or continuous as 1 SD/1 mg/dl increase in uric acid level (unadjusted: RR = 1.73, 95% CI 1.46∼2.06 for categorical data, RR = 1.22, 95% CI 1.03∼1.45 for a 1 SD increase; adjusted: RR = 1.48, 95% CI 1.33∼1.65 for categorical data, RR = 1.15, 95% CI 1.06∼1.26 for a 1 mg/dl increase), and the risk is consistent in subgroup analyses and have a dose-response relationship.

**Conclusions:**

Hyperuricemia may modestly increase the risk of hypertension incidence, consistent with a dose-response relationship.

## Introduction

Hyperuricemia is a metabolic problem that has become increasingly common worldwide and its association with hypertension has been observed for over 130 years [1]. Although lots of observational studies were conducted to investigate the association between uric acid and hypertension, controversy remains. For example, elevated uric acid levels are often associated with established traditional cardiovascular risk factors,it is not quite sure whether uric acid is the cause or consequence of hypertension; studies indicating uric acid as an independent risk factor did not sufficiently control for other known risk factors; how uric acid causes hypertension is not fully understood.

Several events have led to the reappraisal of the role of uric acid in hypertension. Studies using animal models and cell cultures have identified mechanisms by which uric acid might induce hypertension via reducing nitric oxide, activation of renin-angiotensin system, causing smooth muscle cell proliferation and production of various inflammatory mediators PPT-5 [2]–[7]. Some prospective cohort studies that have controlled for multiple risk factors suggest that uric acid may be an independent risk factor for hypertension development and preliminary clinical trials reported benefits from lowing uric acid in HBP patients, but some have inconsistent conclusions [8]–[10].

Previous meta-analysis indicated that high serum uric acid (SUA) levels increased the risk of hypertension incidence [11]. But only prospective cohort studies were included and searched before April 2010. Ever since then, some new high-quality studies assessing the association between uric acid and incident hypertension had been published [12]–[15]. Therefore, we collected all relevant studies to systematically review the association between uric acid and hypertension in order to clarify whether uric acid is an independent risk factor of hypertension.

## Methods

### Search strategy

We conducted a comprehensive literature search of MEDLINE, EMBASE and CBM (Chinese biomedical literature database) through September 2013 using terms of uric acid, urate, hyperuricemia, hypertension, high blood pressure. The following search strategy was used for MEDLINE: (exp Uric Acid/or exp Hyperuricemia/or urate.ab,ti. or uric acid.ab,ti. or Hyperuricemia.ab,ti. or Hyperuric$.ab,ti.) and (exp Hypertension/or hypertension.ab,ti. or high blood pressure.ab,ti.). Similar search strategies were used for EMBASE and CBM. Searches were restricted to English and Chinese publications and human studies. In addition, we searched the reference lists of all identified relevant studies. Our systematic review and meta-analysis was conducted fundamentally according to the checklist of Meta-analysis of Observational Studies in Epidemiology (MOOSE) [16], and the Preferred Reporting Items for Systematic reviews and Meta-Analyses guidelines(PRISMA) [17] (Checklist S1).

### Selection criteria

Two reviewers (JW, TQ) independently screened the titles and abstracts of the studies from the electronic databases to identify all potential eligible studies. Any uncertainties or discrepancies between the two reviewers were resolved through consensus after rechecking the source data and consultation with the third reviewer (JL). We only included cohort studies (both prospective and retrospective) and nested case-control studies investigating the impact of uric acid level on the incident hypertension with a minimum of follow-up duration of 1 year and with a sample size of at least 100 subjects. Included studies must have data on risk ratios (RR) or odds ratios (OR) or hazard ratios (HR) and their corresponding 95% confidence interval (or data to calculate them) of the association between uric acid and hypertension. We excluded cross-sectional studies, literature reviews, and clinical trials relevant to uric acid level lowering in hypertensive patients. We only included the report with most recently updated data when two or more reports were conducted based on the same cohort participants.

### Data extraction

Data were extracted independently by 2 authors (JC, YL) using a standardized data extraction form. Any disagreement was resolved by discussion or in consultation with a third reviewer (JL). For each included study on incident hypertension, we extracted information on title, authors, publication year, country, study design, sample size, study population, age, definition of hyperuricemia, duration of follow-up, number of incident hypertension cases, definition of hypertension, effect size (unadjusted or adjusted RR, OR, HR based on tertiles/quartiles/quintiles levels as categories for uric acid levels or 1 mg/dl/1 SD increase of uric acid levels as continuous data), adjusted confounding variables. For studies that reported several multivariable adjusted RRs, we extracted the one that was most fully adjusted for potential confounders.

### Quality assessment

Two authors (JW, TQ) independently graded the methodological quality of each included study using the Newcastle-Ottawa Scale for cohort study and case-control study [18]. Any disagreement was resolved by discussion or in consultation with a third reviewer (JL). A quality score was calculated based on three broad perspectives: the selection of the study groups (0–4 points), the comparability of the groups (0–2 points), and the ascertainment of either the exposure or outcome of interest (0–3 points) for case-control or cohort studies respectively. As a result, the maximum score was 9 and the minimum score was 0. A higher score represents better methodological quality.

### Statistical analysis

Some studies included in the meta-analysis used the International System of Units to report levels of SUA, so we converted those to the conventional units, using a conversion rate of 16.81 (1 mg/dl = 59.48 µmol/l). We used both of the unadjusted and multivariable-adjusted RR or OR or HR for categories (highest versus lowest categories) and continuous data (1 SD or 1 mg/dl increase in SUA) reported in the original articles to estimate the associations between uric acid levels and hypertension. If these effect sizes above were not available, we used the original data reported in the studies to calculate the unadjusted risk ratios. As the incidence of hypertension and cardiovascular events were sufficiently rare, odds ratios could be assumed to be accurate estimates of risk ratios [19]. We therefore used RRs as the common measure of association across studies. For studies that only provided the data of specific subgroups, such as men and women, we calculated the overall RRs using the data of each subgroup. We converted these values in each study by using their natural logarithms and calculated the standard errors (SEs) from these logarithmic numbers and their corresponding 95% confidence intervals (CIs). We used the *metan* command in Stata to pool the lnRR across studies using the random-effects model as described by DerSimonian and Laird [20], which takes both within-study and between-study variability into account, and to calculate the summary RR estimates and corresponding 95% CI for incidence of hypertension.

We estimated dose-response associations of uric acid levels between incident hypertension based on data from studies reported at least 3 categories of uric acid levels. We pooled the effect sizes and 95% CIs of the development of hypertension for each category compared with the lowest category as reference.

Heterogeneity among studies was assessed using the Cochran's Q test with significance set at *P*<0.10 and quantified with the *I^2^* statistic. Subgroup analyses by sex, ethnicity (Asian or non-Asian), sample size (<3000 or ≥3000) and follow-up duration (<5years or ≥5years) were conducted to assess heterogeneity using multivariate-adjusted RRs of hypertension incidence reported by the original articles. If the data of male/female and total were both available in one study, we selected the male/female data for subgroup analyses by sex, otherwise total data.

The possibility of publication bias was assessed based on the adjusted categorical and continuous data for incident hypertension by the combined method of the Egger regression asymmetry test [21] and visual inspection of funnel plot. We also performed the “trim and fill” method [22] to correct funnel plot asymmetry by simulating the hypothetical “missing” studies possibly arising from publication bias and to derive an adjusted RR by performing a meta-analysis including the filled studies. Agreement between the before and after “trim and fill” RR provides confidence that the results are robust to possible publication bias. Finally, for categorical data and continuous data respectively, we computed the fail-safe number, number of non-significant studies that would bring the *P-value* to non-significant, using 0.05 as the set criterion.

Meta-analysis was performed using Stata 12.0 (StataCorp, College Station, TX). The *metan*, *metabias*, and *metatrim* commands were used for all statistical analysis. Two-sided *P* values below 0.05 were considered statistically significant.

## Results

### Selection and characteristics of studies

**Figure 1:**
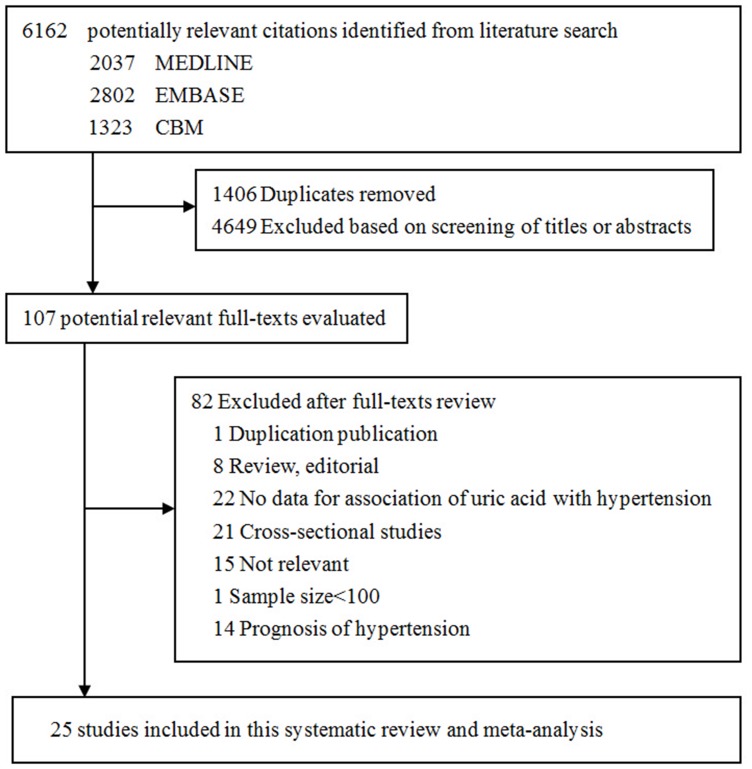
Flowchart of study selection.

A total of 6162 publications were identified (Figure 1). After removing duplicates we excluded 4649 citations based on the screening of titles and abstracts and excluded 82 citations after detailed assessment of the full text. Finally, we identified 25 studies [9], [12]–[15], [23]–[42] that met our inclusion criteria.

Table 1 and S1 shows the characteristics and effect sizes of the 25 studies of uric acid level and risk of hypertension. There were 22 cohort studies, 3 nested case-control studies [9], [25], [41]. Thirteen studies [9], [15], [24]–[26], [29], [30], [36]–[41] were conducted in America, 4 [28], [31], [34], [35] in Japan, 6 [12]–[14], [32], [33], [42] in China, 1 [23] in Israel and 1 [27] in Italy. There were 97,824 study participants (140–25,474 participants), aged 18–89 years old. The lengths of follow up duration varied from 2 to 21.5 years. A total of 23 studies [9], [12]–[15], [25]–[42] adjusted the impact of confounders when assessing the association of uric acid with the risk of hypertension. Hyperuricemia was defined as SUA levels ranged from 5.0 to 7.7 mg/dl in men and from 4.6 to 6.6 mg/dl in women based on the definition reported in the original studies. Seven studies [23]–[25], [28], [30], [31], [38] defined incident hypertension as systolic blood pressure ≥160 mm Hg, diastolic blood pressure ≥95 mm Hg; 18 studies defined incident hypertension as systolic blood pressure ≥140 mm Hg, diastolic blood pressure ≥90 mm Hg, or use of antihypertensive medications at any of the follow-up visits. The quality score varied from 6 to 9 points.

**Table 1:** Characteristics of studies (n = 25) on uric acid levels and incident hypertension.

Author, year, country	Study design	Study population (% men)	Age (years)	Hyperuricemia definition(mg/dl)	Follow up (years)	Total No. of hypertension	Hypertension definition (mmHg)	Study quality*
Kahn 1972, Israel [23]	PC	3829 (100)	≥40	>5.0	5	196	≥160/95	6
Fessel 1980, USA [24]	PC	304	Not mentioned	SUA levels elevated beyond 2 SD from the mean for sex and age	9	29	≥160/95	6
Selby 1990, USA [25]	NC	2062 (39.3)	40.4 for cases, 40.3 for controls	5 quintiles without exact number	9	1031	≥160/95	9
Hunt 1991, USA [26]	PC	1482 (97.3)	Mean: 34.42	SUA levels elevated beyond 2 SD from the mean	7	40	on antihypertensive drugs	7
Jossa 1994, Italy (abstract) [27]	PC	547 (100)	—	—	12	—	≥140/90 or on antihypertensive drugs	—
Nakanishi 1998, Japan [28]	PC	1089 (100)	30–54	≥7.0	6	69	≥160/95	9
Dyer 1999, USA [29]	PC	5115 (43.1)	18–30	1 SD increase from the mean	10	396	≥140/90	9
Imazu 2001, USA [30]	PC	140 (35.7)	40–69	≥6.0	15	17	≥160/95	7
Taniguchi 2001, Japan [31]	PC	6356 (100)	35–60	≥6.2	5–16	639	≥160/95	9
Yeh 2001, Taiwan [32]	PC	2374 (41.4)	>20	1 SD increase	3.23	210	≥140/90	8
Zhang 2001, China [33]	PC	1480 (41.1)	35–59	1 SD increase (1.14 mg/dl)	4	194	≥140/90	8
Nakanishi 2003, Japan [34]	PC	2310 (100)	35–59	≥6.7	6	906	≥140/90 or on antihypertensive drugs	9
Nagahama 2004, Japan [35]	PC	4489 (65.2)	18–89	Men: ≥7.0 Women: ≥6.0	3	289	≥140/90	8
Sundstrom 2005, USA [36]	PC	3329 (44.4)	Mean 48.7	1 SD increase of SUA	4	458	≥140/90	8
Mellen 2006, USA [37]	PC	9104 (45.5)	53.3 (45–64)	≥7.0	9	2561	≥140/90	8
Perlstein 2006, USA [38]	PC	2062 (100)	21–80	≥7.0	Mean: 21.5	892	≥160/95	9
Shankar 2006, USA [39]	PC	2520 (43.7)	43–84	≥6.6	10	956	≥140/90 or on antihypertensive drugs	9
Forman 2007, USA [9]	NC	1454 (100)	61(47–81)	≥6.8	8	745	Medical record review	8
Krishnan 2007, USA [40]	RC	3073 (100)	35–57	≥7.0	6	1569	≥140/90	8
Forman 2009, USA [41]	NC	1496 (0)	32–52	≥4.6	8	748	Questionnaire	8
Zhang 2009, China [42]	PC	7220 (73.8)	Mean: 37.1	Men:≥5.7 Women: ≥4.8	4	1578	≥140/90	9
Wu 2010 China [12]	PC	25474 (79.2)	Not mentioned	≥5.6	2	8358	≥140/90	8
Chien 2011 Taiwan [13]	PC	2506 (49.2)	≥35	≥6.5	6.15	1029	≥140/90	9
Yang 2012 Taiwan [14]	PC	3257 (45.4)	Mean: 37.83	Men:>7.7 Women:>6.6	Mean: 5.41	496	≥140/90, or on antihypertensive drugs	9
Gaffo 2013 USA [15]	RC	4752 (44.9)	18–30	≥6.8	20	—	≥140/90 or on antihypertensive drugs	9

*The quality of each included study was assessed by the Newcastle-Ottawa Scale.

PC: prospective cohort, NC: nested case-control, RC: retrospective cohort, SUA: Serum uric acid, SD: standard deviations, HBP: high blood pressure, SBP: systolic blood pressure, DBP: diastolic blood pressure.

Table S2 shows the effect sizes of hypertension according to the uric acid levels for 11 studies [9], [12], [14], [15], [25], [28], [31], [34], [39], [41], [42] that provided at least 3 categories of uric acid levels.

### Uric acid and the incidence of hypertension

Eleven studies [9], [12], [23]–[25], [28], [29], [34]–[36], [40] provided unadjusted relative risk for hypertension incidence, of which 10 studies [9], [12], [23]–[25], [28], [34]–[36], [40] provided 11 sets categorical data and 3 studies [9], [29], [36] provided 6 sets continuous data (1 SD increase). There was significant heterogeneity among studies of both categorical (*I^2^* = 89.1%, *P*<0.001) and continuous data (*I^2^* = 90.2%, *P*<0.001). Random-effects model meta-analysis showed that hyperuricemia in terms of both categorical and continuous data could significantly increase the risk of hypertension development (RR = 1.73, 95% CI 1.46∼2.06; RR = 1.22, 95% CI 1.03∼1.45, respectively).

**Figure 2:**
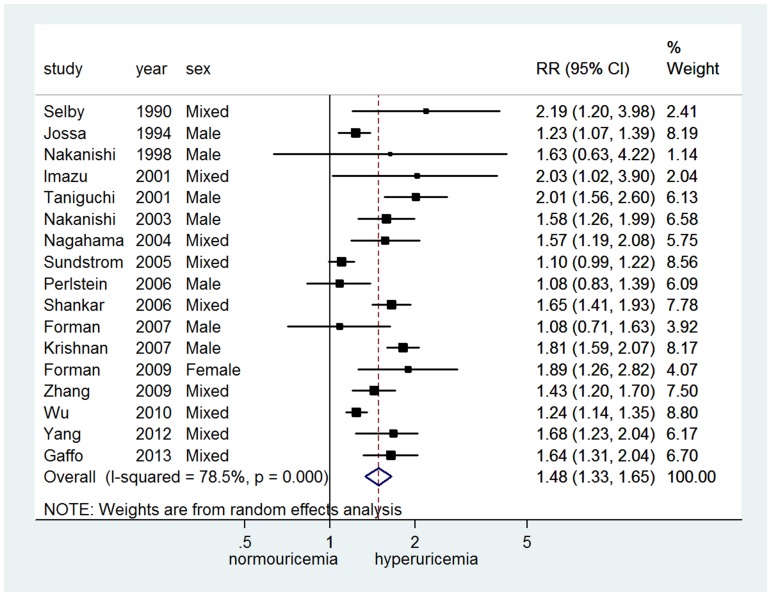
Adjusted relative risk of uric acid level and incident hypertension (categorical data).

**Figure 3:**
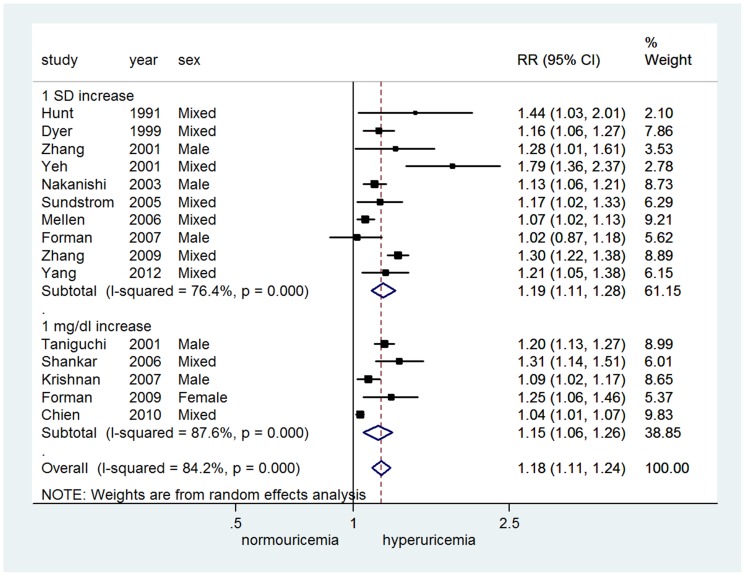
Adjusted relative risk of uric acid level and incident hypertension (continuous data).

Twenty-three studies [9], [12]–[15], [25]–[42] provided adjusted relative risk for hypertension incidence, of which 17 studies [9], [12], [14], [15], [25], [27], [28], [30], [31], [34]–[36], [38]–[42] provided 24 sets categorical data and 15 studies provided 25 sets continuous data (5 studies [13], [31], [39]–[41] set as 1 mg/dl increase and 10 studies [9], [14], [26], [29], [32]–[34], [36], [37], [42] set as 1 SD increase ranged from 0.9 to 1.5 mg/dl). There was significant heterogeneity among studies of both categorical (*I^2^* = 78.5%, *P*<0.001) and continuous data *(I^2^* = 84.2%, *P*<0.001). Random-effects model meta-analysis showed that hyperuricemia in terms of categorical data could significantly increase the risk of hypertension development (RR = 1.48, 95% CI 1.33∼1.65) (Figure 2). Similarly, the risk of incident hypertension increased by 15% (RR = 1.15, 95% CI 1.06∼1.26) for every 1 mg/dl increase in SUA and 19% (RR = 1.19, 95% CI 1.11∼1.28) for every 1 SD increase (Figure 3).

### Subgroup analysis

To explore the between-study heterogeneity, we performed subgroup analyses by sex, ethnicity (Asian or non-Asian), duration of follow-up (<5 years or ≥5 years) and sample size (<3000 or ≥3000) in both adjusted categorical and continuous data. The finding of increased hypertension risk in hyperuricemia was consistently found in all of the subgroup analyses (Table 2).

**Table 2:** Subgroup meta-analysis of uric acid levels and adjusted risk of incident hypertension*.

Subgroup	No. of included studies	Heterogeneity test(*I* ^2^, *P*)	Summary RR (95% CI)
**Categorical data**			
Sex			
Female	6	0.0%, *P = 0.770*	1.66 (1.46–1.88)
Male	12	68.3%, *P<0.001*	1.49 (1.31–1.68)
Ethnicity			
Asian	7	68.8%, *P = 0.004*	1.53 (1.32–1.78)
Non-Asian	10	83.7%, *P<0.001*	1.45 (1.23–1.72)
Follow-up			
<5 years	4	70.7%, *P = 0.017*	1.27 (1.12–1.45)
≥5 years	13	66.5%, *P<0.001*	1.57 (1.39–1.78)
Sample size			
<3000	9	60.0%, *P = 0.010*	1.45 (1.24–1.70)
≥3000	8	87.1%, *P<0.001*	1.51 (1.29–1.76)
**Continuous data**			
Sex			
Female	5	21.4%, *P = 0.278*	1.12 (1.05–1.19)
Male	9	64.9%, *P = 0.004*	1.15 (1.08–1.21)
Ethnicity			
Asian	7	91.8%, *P<0.001*	1.22 (1.10–1.34)
Non-Asian	8	52.4%, *P = 0.040*	1.14 (1.08–1.21)
Follow-up			
<5 years	4	59.9%, *P = 0.058*	1.31 (1.16–1.48)
≥5 years	11	76.7%, *P<0.001*	1.14 (1.08–1.19)
Sample size			
<3000	8	81.8%, *P<0.001*	1.20(1.09–1.32)
≥3000	7	78.2%, *P<0.001*	1.17 (1.10–1.24)

*The variables adjusted in each primary study were shown in Table S1.

### Dose-response association

**Figure 4:**
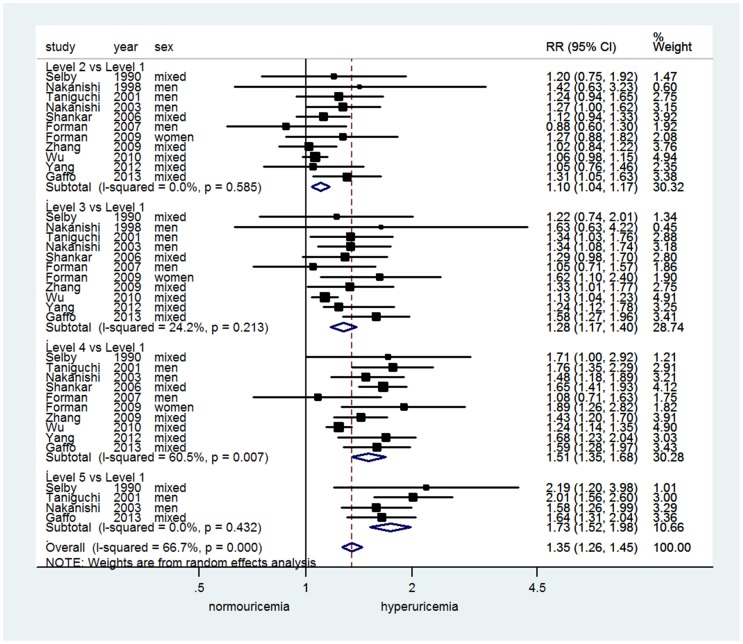
Dose-response associations of uric acid levels on incident hypertension.

To explore whether there were dose-response associations of uric acid levels on incident hypertension, we used the data from studies reported at least 3 categories of uric acid levels and pooled the RRs of the development of hypertension for each category compared with the lowest category as reference. Random effects meta-analyses showed that higher categories of uric acid were associated with higher risk of hypertension development (Figure 4).

### Publication bias

**Figure 5:**
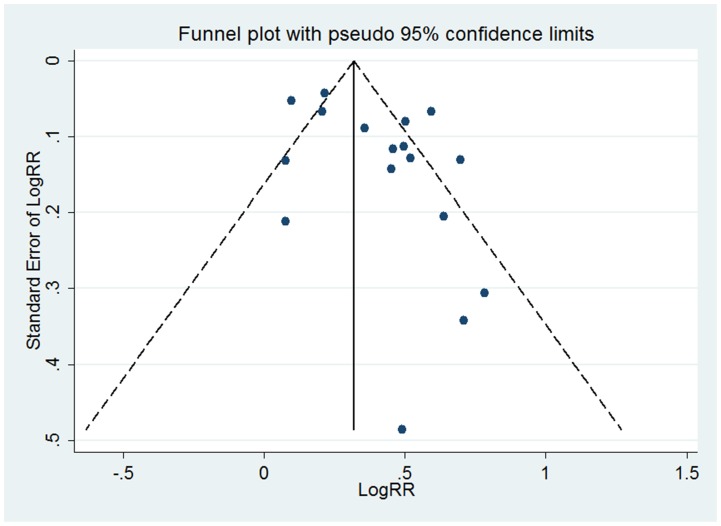
Funnel plot of studies reporting categorical data of hyperuricemia and risk of incident hypertension.

**Figure 6:**
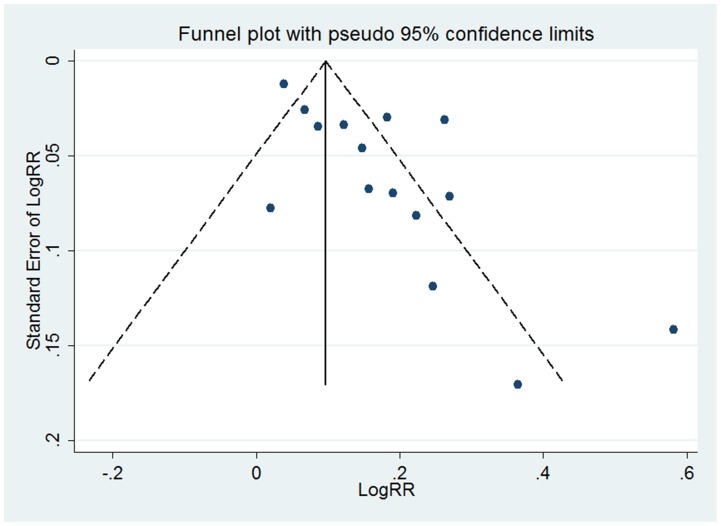
Funnel plot of studies reporting continuous data of hyperuricemia and risk of incident hypertension.

Visual inspection of the funnel plots on 20 sets of adjusted categorical data from 17 studies and 19 sets of adjusted continuous data from 15 studies of uric acid level and risk of incident hypertension revealed asymmetrical, indicating potential risk of publication bias (Figure 5–6). Therefore, we undertook a sensitivity analysis using the trim and fill method, which conservatively imputes hypothetical negative unpublished studies to mirror the positive studies that cause funnel plot asymmetry. Two imputed studies for categorical data and five for continuous data were needed to produce symmetrical funnel plots. The pooled analysis incorporating the hypothetical studies continued to show a statistically significant association between hyperuricemia and incident hypertension (RR = 1.40, 95% CI 1.27–1.54 for categorical data; RR = 1.16, 95% CI 1.10–1.22 for continuous data). The fail-safe number was 1283 for categorical data and 886 for continuous data,indicating that 1283 “negative” studies for categorical data and 886 for continuous data would be needed to increase the P value for the meta-analysis to above 0.05.

## Discussion

Our meta-analysis of both unadjusted and adjusted data, categorical and continuous data showed that hyperuricemia increased the risk of incident hypertension. The risk of incident hypertension appeared to increase with increasing uric acid level. Furthermore, the association was consistent across subgroups. Our results confirm that hyperuricemia is an independent risk factor of hypertension development.

Recent experimental and clinical studies suggest that an elevated SUA level may lead to hypertension. An animal model of mild hyperuricemia induced by the administration of an uricase inhibitor provided the first direct evidence that elevated uric acid may lead to blood pressure elevation [5]. The potential mechanism involves two-step process. The early hyperuricemia caused renal vasoconstriction mediated by endothelial dysfunction resulting from a reduction in endothelial levels of nitric oxide and activation of the rennin-angiotensin system [5], [7], followed by progressive renal microvascular disease resulting from uric acid induced cellular proliferation after urate enters into the vascular smooth muscle, inflammation due to production of various inflammatory mediators including C-reactive protein and monocyte chemoattractant protein-1, oxidative stress and activation of the local rennin-angiotensin system [43]. The results are that early hypertension is salt-resistant in that it occurs even in the presence of a low-salt diet and responds to lowering of uric acid [5], after sufficient narrowing of the arteriolar lumen occurs, hypertension becomes salt-driven, renal-dependent, and independent of uric acid levels [44]. Preliminary clinical trials also support a key role for uric acid in the pathogenesis of early onset essential hypertension. An open-label pilot study was conducted in 5 children with newly diagnosed, untreated essential hypertension [45]. They were treated with allopurinol for 4 weeks followed by a 6-week washout period. All 5 children had substantial drops in casual blood pressure and 4 of 5 subjects developed normal blood pressure by ambulatory blood pressure monitoring criteria. However, 6 weeks after discontinuing the allopurinol, the blood pressure of all 5 children rebounded to baseline levels. The findings of this pilot study should be interpreted with caution because no placebo group was included. Another randomized, double-blind, placebo-controlled, cross-over trial was conducted in 30 adolescents with newly diagnosed, never-treated stage 1 essential hypertension and hyperuricemia [46]. Twenty of the 30 patients achieved normal blood pressure in both casual and ambulatory criteria while taking allopurinol vs. 1 patients while taking placebo (P<0.001).

Our results are consistent with a previous systematic review by Grayson *et al*. [11], which included 55,607 participants from 18 prospective cohort studies representing that the adjusted risk ratio for incident hypertension was 1.41 (95% CI 1.23∼1.58) in hyperuricemia. However, only prospective cohort studies and a handful of Asian studies (4/18) were included and searched before April 2010 in this systematic review. Ever since then, some new high-quality studies assessing the association between uric acid and incident hypertension had been published. We, therefore, conducted an updated systematic review and meta-analysis to obtain a more precise estimation of the association between hyperuricemia and hypertension. There are some important differences in these two reviews. First, we conducted an updated and comprehensive literature search including Chinese database. As a result, we included more studies, especially Asian studies (10/25), not only prospective cohort studies but also retrospective cohort studies and nested case-control studies. Consequently, our results should be more applicable. Second, as some primary studies examined the relationship between different serum uric acid levels and risk of hypertension incidence, we estimated dose-response associations of uric acid levels with incident hypertension and the results showed that higher categories of uric acid were associated with higher risk of hypertension development. Since the demonstration of a dose-response relationship in an observational study supporting a causal explanation of a disease-exposure association, our results should be more reliable.

### Strengths and Limitations

Our study has several strengths. First, temporality: Of the 25 included studies, 22 were cohort studies and three were nested case-control studies, which greatly reduces the likelihood of selection bias and denotes that an elevated uric acid precedes the development of hypertension. Second, dose-response relationship: Of the 25 studies assessed the association of uric acid level with the development of hypertension, 11 studies [14], [15], [23], [25], [27], [30], [34], [35], [39], [41], [42] found that higher categories of uric acid were associated with higher risk of hypertension development. In addition, 13 studies found that the association of SUA level to the future hypertension was continuous and dose-dependent. Third, the relationship of SUA with the development of hypertension is consistent regardless of whether other traditional risk factors for hypertension development were adjusted or not and whether how many and what kinds of risk factor were adjusted. Fourth, the consistency of the association between SUA level and hypertension development across multiple categories and subgroup analyses indicate that our conclusions in the meta-analysis were not dependent on arbitrary decisions.

Some limitations should be noted in our meta-analysis. First, as shown in Table S1, although most included studies adjusted confounding factors relevant to hypertension more or less including demographic and lifestyle factors, baseline blood pressure, body mass index (BMI), renal function, lipid levels, the number and types of adjusted factors are different. There are maybe residual confounding factors which have effects on the findings. However, the pooled estimates found that no matter confounding factors adjusted or unadjusted,no matter how many confounding factors were adjusted, the conclusion that hyperuricemia is a risk factor of hypertension development is consistent, which reduce the likelihood that residual confounding can fully explain the findings and the causal effect of uric acid on hypertension. Second, the included studies had potential risk of bias due to differences in the representativeness of cohort, definition of hyperuricemia, completeness report of follow-up and definition of incident hypertension. Third, our meta-analysis only included studies published in English and Chinese, and did not searching for unpublished studies that might contribute to the asymmetrical funnel plot. However, the trim and fill method and sensitivity analysis incorporating the hypothetical unpublished negative studies did not change the association between hyperuricemia and incident hypertension and implies the robust of our findings.

### Heterogeneity in included studies

It is not surprised that there was significant heterogeneity among the included studies given the substantial differences in study populations such as age, sex ratio, baseline characteristics, definition of hyperuricemia and hypertension, follow-up durations, drop-out and risk of bias. Further evaluation of effect modification of these differences on the association of uric acid and hypertension is needed in larger studies of high quality or individual patient data meta-analysis that has more power to detect effect modification than our study-level meta-analysis.

## Conclusions

Our review shows that uric acid elevation is consistently associated with high risk of incident hypertension. Although systematic review and meta-analysis of epidemiologic studies cannot establish causality of uric acid as a causal factor in hypertension, the consistency of the association across diverse populations, the dose-response association, and the supporting evidence from both animal models and preliminary clinical trial in human indicate that uric acid may play an important role in hypertension. Future researches, particularly high-quality randomized controlled trials, are needed to verify whether reducing the serum uric acid level could be beneficial for hypertension prevention and treatment, and to provide basis for the reasonable application of uric acid lowering drugs.

## Supporting Information

Table S1 **Effect sizes of hypertension according to uric acid levels in 25 included studies.**
(DOCX)

Table S2 **Effect sizes of hypertension according to the uric acid levels for studies that provided at least 3 categories of uric acid levels.**
(DOCX)

Checklist S1 **PRISMA Checklist.**
(DOC)
