# Supplementary material for: Hyperuricemia and Risk of Incident Hypertension: A Systematic Review and Meta-Analysis of Observational Studies
Source: PLoS One. 2014 Dec 1;9(12):e114259. doi: 10.1371/journal.pone.0114259 (PMC4250178; doi:10.1371/journal.pone.0114259)
Supplement: Table S1 — Effect sizes of hypertension according to uric acid levels in 25 included studies. (DOCX) [file pone.0114259.s001.docx]

**Table S1-****Effect sizes of hypertension according to uric acid levels in 25 included studies**

| **Study** | **Comparison** | **Model** | **Effect sizes(95% CI)** | **Adjustment for covariates** |
| --- | --- | --- | --- | --- |
| Kahn 1972, Israel[23] | Highest vs lowest tertile | Crude | RR for male: 1.84(1.32-2.57) | NA |
| Fessel 1980, USA[[24](#_ENREF_24)] | Hyperuricemia vs normouricemia | Crude | RR:10.87(3.88-30.42) | NA |
| Selby 1990, USA[[25](#_ENREF_25)] | Highest vs lowest quintile | Crude | OR: 2.99(2.06-4.34) | NA |
|  | Highest vs lowest quintile | Multivariable | OR: 2.19(1.20-3.98) | BMI, history of HBP, alcohol consumption, heavy salty use, baseline SBP, DBP |
| Hunt 1991, USA[[26](#_ENREF_26)] | 1 SD difference  (1.16 mg/dl) | Multivariable | RR: 1.44(1.03-2.01) | Age, sex, BMI, SBP, DBP, history of HBP, height, weight, TG |
| Jossa 1994, Italy[[27](#_ENREF_27)] | Highest vs lowest quintile | Multivariable | RR: 1.23(1.07-1.39) | Age, BMI, cholesterol, TG |
| Nakanishi 1998, Japan[[28](#_ENREF_28)] | Highest vs lowest tertile | Crude | RR: 1.91(0.81-4.50) | NA |
|  | Highest vs lowest tertile | Multivariable | HR: 1.63(0.63-4.22) | Multivariate hazard ratio by Cox proportional hazards model |
| Dyer 1999, USA[[29](#_ENREF_29)] | 1 SD increase from the mean (1.2 mg/dl for black men, 1.1 mg/dl for white men, 1.0 mg/dl for black women, 0.9 mg/dl for white women) | Crude | OR for men:  Black: 1.31(1.14-1.50)  White:1.50 (1.26-1.79)  OR for women:  Black: 1.40(1.23-1.61)  White: 1.23(0.96-1.57) | NA |
|  | 1 SD increase from the mean (1.2 mg/dl for black men, 1.1 mg/dl for white men, 1.0 mg/dl for black women, 0.9 mg/dl for white women) | Multivariable | OR for men:  Black:1.21(1.03-1.41)  White:1.16(0.96-1.40)  OR for women:  Black:1.14(0.97-1.33)  White:1.04(0.79-1.37) | Age, SBP, waist circumference, physical activity, alcohol intake, pulse, smoking, education, insulin, TG, HDL-cholesterol |
| Imazu 2001, USA[[30](#_ENREF_30)] | Quartile 4(≥6.0 mg/dl) vs Quartile 1-3 | Multivariable | RR:2.03(1.02-3.90) | Age, sex, BMI, glucose, insulin, lipids and baseline SBP |
| Taniguchi 2001, Japan[[31](#_ENREF_31)] | Highest vs lowest quintile | Multivariable | RR: 2.01(1.56-2.60) | Age, BMI, alcohol consumption, smoking, physical activity, duration of the walk to work, FPG |
|  | 1 mg/dl increase | Multivariable | RR: 1.20(1.13-1.27) |  |
| Yeh 2001, Taiwan[[32](#_ENREF_32)] | 1 SD increase  (1.5 mg/dl for men,  1.3 mg/dl for women) | Multivariable | HR for men:  1.94(1.39-2.69)  HR for women:  1.49(0.89-2.47) | Age, baseline SBP, BMI, glucose concentration, alcohol intake, TG, glucose |
| Zhang 2001, China[[33](#_ENREF_33)] | 1 SD increase  (1.14 mg/dl) | Multivariable | OR for men:  1.28(1.01-1.61) | Age, BMI, smoking, alcohol intake, baseline SBP |
| Nakanishi 2003, Japan[[34](#_ENREF_34)] | Highest vs lowest quintile | Crude | HR: 1.93(1.61-2.30) | NA |
|  |  | Multivariable | HR: 1.58(1.26-1.99) | Age, BMI, history of hypertension, smoking, alcohol intake, regular physical exercise, MBP, FPG, TC, HDL, TG at study entry |
|  | 1 SD increase  (1.3 mg/dl) | Multivariable | HR: 1.13(1.06-1.21) |  |
| Nagahama 2004, Japan[[35](#_ENREF_35)] | Hyperuricemia vs normouricemia | Crude | OR for men:  1.46(1.09-1.96)  OR for women:  2.42(1.36-4.28) | NA |
|  | Hyperuricemia vs normouricemia | Multivariable | OR for men:  1.49(1.09-2.03)  OR for women:  1.94(1.05-3.57) | Age, history of hypertension, alcohol intake, smoking, obesity, high TC and TG, low-HDL, DM |
| Sundstrom 2005, USA[[36](#_ENREF_36)] | 1 SD increase(1.3 mg/dl) | Crude | OR: 1.29(1.17-1.42) | NA |
|  | Highest vs lowest quartile | Crude | OR: 1.23(1.13-1.35) |  |
|  | 1 SD increase(1.3 mg/dl) | Multivariable | OR: 1.17(1.02-1.33) | Age, sex, BMI, DM, smoking, alcohol intake, Cr, proteinuria, GFR, baseline BP and interim weight changes, baseline use of cardiac medication |
|  | Highest vs lowest quartile | Multivariable | OR: 1.10(0.99-1.22) |  |
| Mellen 2006, USA[[37](#_ENREF_37)] | 1 SD increase(1.4mg/dl for black men, 1.3 mg/dl for black women, 1.2 mg/dl for white men, 1.1 mg/dl for white women) | Multivariable | HR for overall cohort:  1.07(1.02-1.13)  HR for men:  Black: 1.32(1.14-1.54)  White:1.01(0.94-1.09)  HR for women:  Black:1.16(1.03-1.31)  White:1.04(0.96-1.11) | Age, race, sex, baseline BP, BMI, renal function, DM, smoking, estimated GFR |
| Perlstein 2006, USA[[38](#_ENREF_38)] | Hyperuricemia vs normouricemia | Multivariable | RR: 1.08(0.83-1.39) | Age, SBP, DBP, BMI, abdominal circumference, smoking, alcohol, TG, TC and plasma glucose |
| Shankar 2006, USA[[39](#_ENREF_39)] | Highest vs lowest quartile | Multivariable | RR for whole cohort:  1.65(1.41-1.93)  RR for men:  1.48(1.17-1.87)  RR for women:  1.71(1.40-2.09) | Age, gender, BMI, education, smoking, alcohol intake, TC, HDL, physical activity, diabetic status, glycosylated haemoglobin, pulse rate, SBP, DBP, and GFR |
|  | 1SD increase in SUA  (1.0 mg/dl) | Multivariable | RR for whole cohort: 1.31(1.14-1.51) |  |
| Forman 2007, USA[[9](#_ENREF_9)] | Highest vs lowest quartile | Crude | RR:1.05(0.79-1.40) | NA |
|  | Highest vs lowest quartile | Multivariable | RR:1.08(0.71-1.63) | BMI, eGFR, alcohol intake, smoking, change in weight, physical activity, race, family history of HBP, baseline SBP and DBP |
|  | 1 SD increase in SUA (1.14 mg/dl) | Crude | RR:1.02(0.92-1.13) | NA |
|  | 1 SD increase in SUA (1.14 mg/dl) | Multivariable | RR:1.02(0.87-1.18) | BMI, eGFR, alcohol intake, smoking, change in weight, physical activity, race, family history of HBP, baseline SBP and DBP |
| Krishnan 2007, USA[[40](#_ENREF_40)] | Hyperuricemia vs no hyperuricemia | Crude | HR:1.6 (1.5-1.8) | NA |
|  | Hyperuricemia vs no hyperuricemia | Multivariable | HR:1.81(1.59-2.07) | Age, SBP DBP, Cr, TC, TG, alcohol use, smoking, proteinuria, BMI, risk factor interventions and use of diuretics |
|  | 1mg/dl increase | Multivariable | HR:1.09(1.02-1.17) |  |
| Forman 2009, USA [[41](#_ENREF_41)] | Highest vs lowest quartile | Multivariable | OR:1.89(1.26-2.82) | Age, race, date of blood sample collection, day of menstrual cycle if premenopausal, and time of day of the blood collection, BMI, smoking, physical activity, alcohol intake, family history of HBP, eGFR, TC, TG, insulin, homocysteine, sICAM-1 |
|  | 1mg/dl increase | Multivariable | OR:1.25(1.06-1.46) |  |
| Zhang 2009, China[[42](#_ENREF_42)] | Highest vs lowest quartile | Multivariable | RR for men:  1.39(1.16-1.68)  RR for women:  1.85(1.06-3.24) | Age, BMI, smoking, alcohol intake, physical activity, blood glucose, TG, TC, HDL, Cr, GRF, proteinuria, salt consumption, baseline SBP and DBP, family history of hypertnesion, waist circumference |
|  | 1 SD increase in SUA  (1.2 mg/dl) | Multivariable | RR: 1.30(1.22-1.38) |  |
| Wu 2010  China[[12](#_ENREF_12)] | Highest vs lowest quartile | Crude | RR:1.83(1.69-1.97) | NA |
|  | Highest vs lowest quartile | Multivariable | RR:1.24(1.14-1.35) | Age, sex, BMI, smoking, alcohol intake, baseline SBP and DBP |
| Chien 2011  Taiwan[[13](#_ENREF_13)] | 1 mg/dl increase | Multivariable | RR:1.039(1.014-1.065) | Sex, age, BMI, SBP, DBP, WBC, fasting glucose |
| Yang 2012  Taiwan[[14](#_ENREF_14)] | Highest vs lowest quartile | Multivariable | HR for total  1.68(1.23-2.04)  HR for men  1.41(1.08-1.92)  HR for women  1.64(1.23-2.03) | Sex (for the total), age, BMI, waist circumference, TC, TG, glucose, SBP, DBP, and serum creatinine levels |
|  | 1 SD increment in SUA | Multivariable | HR for total  1.21(1.05-1.38)  HR for men  1.17(1.02-1.36)  HR for women  1.15(1.04-1.33) |  |
| Gaffo 2013  USA[[15](#_ENREF_15)] | Highest vs lowest quintile | Multivariable | HR for men  2.05(1.47-2.84)  HR for women  1.37(1.02-1.83) | Age, race, BMI, GFR, alcohol intake, physical activity score, insulin level, LDL, HDL, TG, smoking, education |

NA: not applicable, SD: standard deviations, BMI: Body mass index, HBP: high blood pressure, MBP: mean blood pressure, SBP: systolic blood pressure, DBP: diastolic blood pressure, Cr: creatinine, TC: total cholesterol, TG: triglycerides, FPG: fasting plasma glucose, HDL: high-density lipoprotein cholesterol, LDL: low-density lipoprotein cholesterol, DM: diabetes mellitus, GFR: glomerular filtration rate, sICAM: soluble intercellular cell adhesion molecule, WBC: white blood cell count
