# Supplementary material for: Hyperuricemia and Risk of Incident Hypertension: A Systematic Review and Meta-Analysis of Observational Studies
Source: PLoS One. 2014 Dec 1;9(12):e114259. doi: 10.1371/journal.pone.0114259 (PMC4250178; doi:10.1371/journal.pone.0114259)
Supplement: Table S2 — Effect sizes of hypertension according to the uric acid levels for studies that provided at least 3 categories of uric acid levels. (DOCX) [file pone.0114259.s002.docx]

**Table S2-Effect sizes of hypertension according to the uric acid levels for studies that provided at least 3 categories of uric acid levels**

| **Author, year, country** | **Model** | **Categories** | **Effect size(95% CI)** |
| --- | --- | --- | --- |
| Selby 1990, USA[[25](#_ENREF_25)] | Multivariable | Quintile 1 | 1.00 |
|  |  | Quintile 2 | 1.20(0.75-1.92) |
|  |  | Quintile 3 | 1.22(0.74-2.01) |
|  |  | Quintile 4 | 1.71(1.00-2.92) |
|  |  | Quintile 5 | 2.19(1.20-3.98) |
| Nakanishi 1998, Japan[[28](#_ENREF_28)] | Multivariable | ≤4.9 mg/dl | 1.00 |
|  |  | 5.0-6.9 mg/dl | 1.42(0.63-3.23) |
|  |  | ≥7.0 mg/dl | 1.63(0.63-4.22) |
| Taniguchi 2001, Japan[[31](#_ENREF_31)] | Multivariable | 36-250μmol/l | 1.00 |
|  |  | 251-290μmol/l | 1.24(0.94-1.65) |
|  |  | 291-330μmol/l | 1.34(1.03-1.76) |
|  |  | 331-370μmol/l | 1.76(1.35-2.29) |
|  |  | 371-833μmol/l | 2.01(1.56-2.60) |
| Nakanishi 2003, Japan[[34](#_ENREF_34)] | Multivariable | <280μmol/l | 1.00 |
|  |  | 208-320μmol/l | 1.27(1.00-1.62) |
|  |  | 321-350μmol/l | 1.34(1.08-1.74) |
|  |  | 351-397μmol/l | 1.48(1.18-1.89) |
|  |  | ≥398μmol/l | 1.58(1.26-1.99) |
| Shankar 2006, USA[[39](#_ENREF_39)] | Multivariable | ≤260μmol/l | 1.00 |
|  |  | 261-325μmol/l | 1.12(0.94-1.33) |
|  |  | 326-390μmol/l | 1.29(0.98-1.70) |
|  |  | >390μmol/l | 1.65(1.41-1.93) |
| Forman 2007, USA[[9](#_ENREF_9)] | Multivariable | 2.4-5.1 mg/dl | 1.00 |
|  |  | 5.2-5.9 mg/dl | 0.88(0.60-1.30) |
|  |  | 6.0-6.7 mg/dl | 1.05(0.71-1.57) |
|  |  | 6.8-11.5 mg/dl | 1.08(0.71-1.63) |
| Forman 2009, USA [[41](#_ENREF_41)] | Multivariable | 1.5-3.3 mg/dl | 1.00 |
|  |  | 3.4-3.9 mg/dl | 1.27(0.88-1.82) |
|  |  | 4.0-4.5 mg/dl | 1.62(1.10-2.40) |
|  |  | 4.6-8.8 mg/dl | 1.89(1.26-2.82) |
| Zhang 2009, China[[42](#_ENREF_42)] | Multivariable | Men： | |
|  |  | <247μmol/l | 1.00 |
|  |  | 247-293μmol/l | 0.97(0.80-1.18) |
|  |  | 294-337μmol/l | 1.23(0.87-1.73) |
|  |  | ≥338μmol/l | 1.39(1.16-1.68) |
|  |  | Women： | |
|  |  | <213μmol/l | 1.00 |
|  |  | 213-249μmol/l | 1.49(0.85-2.63) |
|  |  | 250-286μmol/l | 1.58(0.93-2.50) |
|  |  | ≥287μmol/l | 1.85(1.06-3.24) |
| Wu 2010，China[[12](#_ENREF_12)] | Multivariable | ≤228μmol/l | 1.00 |
|  |  | 229-277μmol/l | 1.06(0.98-1.15) |
|  |  | 278-331μmol/l | 1.13(1.04-1.23) |
|  |  | ≥332μmol/l | 1.24(1.14-1.35) |
| Yang 2012, Taiwan[[14](#_ENREF_14)] | Multivariable | <5.0 mg/dl | 1.00 |
|  |  | 5.0–6.0 mg/dl | 1.05 (0.76–1.46) |
|  |  | 6.1–7.2 mg/dl | 1.24 (1.12–1.78) |
|  |  | >7.2 mg/dl | 1.68 (1.23–2.04) |
| Gaffo 2013, USA[[15](#_ENREF_15)] | Multivariable | Men： | |
|  |  | <309μmol/l | 1.00 |
|  |  | 309-344μmol/l | 1.27 (0.90-1.80) |
|  |  | 345-380μmol/l | 1.86 (1.34-2.57) |
|  |  | 381-415μmol/l | 1.67 (1.19-2.34) |
|  |  | ≥416μmol/l | 2.05 (1.47-2.84) |
|  |  | Women： | |
|  |  | <214μmol/l | 1.00 |
|  |  | 214-249μmol/l | 1.33 (1.00-1.76) |
|  |  | 250-273μmol/l | 1.38 (1.03-1.85) |
|  |  | 274-314μmol/l | 1.54 (1.17-2.04) |
|  |  | ≥315μmol/l | 1.37 (1.02-1.83) |
